# A case of rapidly progressing sarcomatous intrahepatic cholangiocarcinoma with suddenly appearing lymph node metastasis

**DOI:** 10.1186/s40792-023-01804-7

**Published:** 2024-01-08

**Authors:** Eisho Kanemitsu, Rei Takahashi, Setsuko Nakanishi, Satoru Sueyoshi, Atsushi Kobayashi, Takao Nishimura, Hiromitsu Nagata

**Affiliations:** 1 Department of Surgery, JCHO Yamatokoriyama Hospital, 1-62, Asahi-Cho, Yamatokoriyama, Nara 639-1013 Japan; 2 Graduate School of Pharmaceutical Science, Doshisha Women’s College of Liberal Arts, 97-1, Kodo, Kyotanabe, Kyoto 610-0395 Japan; 3 Department of Radiology, JCHO Yamatokoriyama Hospital, 1-62, Asahi-Cho, Yamatokoriyama, Nara 639-1013 Japan; 4 Department of Radiology, Saiseikai Chuwa Hospital, 323, Abe, Sakurai, Nara 633-0054 Japan

**Keywords:** Intrahepatic cholangiocarcinoma, Sarcomatous pattern, Rapid progression, Tumor growth rate

## Abstract

**Background:**

The sarcomatous variant of carcinoma is relatively rare in intrahepatic cholangiocarcinoma (ICC). Sarcomatous ICC (SICC) is associated with a poorer prognosis compared with ICC. SICC is rarely diagnosed before surgery due to non-descriptive findings; it progresses rapidly, resulting in miserable prognosis. Here, we report a case of rapidly progressing SICC that showed a clinically significant tumor growth rate.

**Case presentation:**

A 77-year-old woman who had undergone ileocecal resection for cecal cancer 5 years previously was found to have elevated levels of the tumor marker carbohydrate antigen 19-9. Although an abdominal computed tomography (CT) scan did not detect any liver mass lesions until 3 months before this serum examination, the subsequent CT scan revealed a hypodensity 20 mm mass lesion in the right anterior section. Contrast-enhanced CT and magnetic resonance imaging revealed peripheral enhancement in the arterial-to-equilibrium phase. Fluorodeoxyglucose positron emission tomography revealed uptake in the lesion. None of the imaging modalities showed lymph node swelling or distant metastases. She underwent hepatectomy under the diagnosis of ICC or an atypical metastasis from previous cecal cancer. Although preoperative images showed no suspicious lymph node metastasis 3 weeks prior, the hilar lymph node swelled 3 cm and contained adenocarcinoma. Consequently, the patient underwent right anterior sectionectomy and lymph node dissection of the hepatoduodenal ligament. Histopathological examination revealed that the liver tumor was a poorly differentiated adenocarcinoma with sarcomatous pattern. While the patient received adjuvant gemcitabine and S-1 therapy, lymph node metastasis appeared in the mediastinum 13 months after the surgery. She received gemcitabine + cisplatin + S-1 therapy but died 20 months after surgery.

**Conclusion:**

SICC and lymph node metastasis clinically appeared within 3 months and 3 weeks, respectively. Suspected ICC that rapidly progresses should be considered SICC and treated with early resection. SICC is often missed in clinical diagnosis and has a poor prognosis, even after curative resection. While an alternative strategy involving preoperative biopsy and neoadjuvant therapy may be beneficial, it should be approached with discretion due to the potential risks of tumor progression and peritoneal dissemination.

## Background

Sarcomatous features of carcinomas have been described at various sites [1–5], and these tumors are called sarcomatous carcinomas or carcinomas with a sarcomatous pattern. In liver tumors, this feature is more frequently seen in hepatocellular carcinoma (HCC) than intrahepatic cholangiocarcinoma (ICC) [6, 7]. As sarcomatous carcinomas are well-known to have a poorer prognosis compared to other carcinomas, radical resection is considered the best therapeutic option for a cure [8]. However, a precise diagnosis is difficult to obtain preoperatively and can be established coincidently by histopathological and immunohistochemical examinations after resection [9]. This dilemma makes preoperative diagnosis time-consuming. The tumor doubling-time is considered to correlate with the tumor growth rate, which depends on the histological type and varies widely with a median value of approximately 2 months [10]. The interval between the patient’s office visit and the commencement of surgery typically takes at least one month, a period that is rarely conducive to curable tumors advancing towards an unresectable state. However, aggressive spreading and a high tendency to metastasize in sarcomatous carcinoma potentially inhibit curative resection when considering the diagnosis and therapeutic options. The present case demonstrates that sarcomatous ICC (SICC) appeared within three months and rapidly induced 3 cm lymph node metastasis within three weeks. This report aims to strengthen the clinicians’ understanding of SICC and reduce the incidence of missing curative resections.

## Case presentation

A 76-year-old Japanese woman was admitted to our hospital with a liver tumor discovered during a follow-up examination of past colorectal cancer. Comorbidities included hypertension and hyperlipidemia, with no history of drinking or smoking. She had undergone ileocecal resection for cecal cancer (T1N0M0, according to the Union for International Cancer Control (UICC) classification) 5 years and 3 months ago. Physical examination revealed no abnormal findings, except for a lateral rectus incision.

**Fig. 1:**
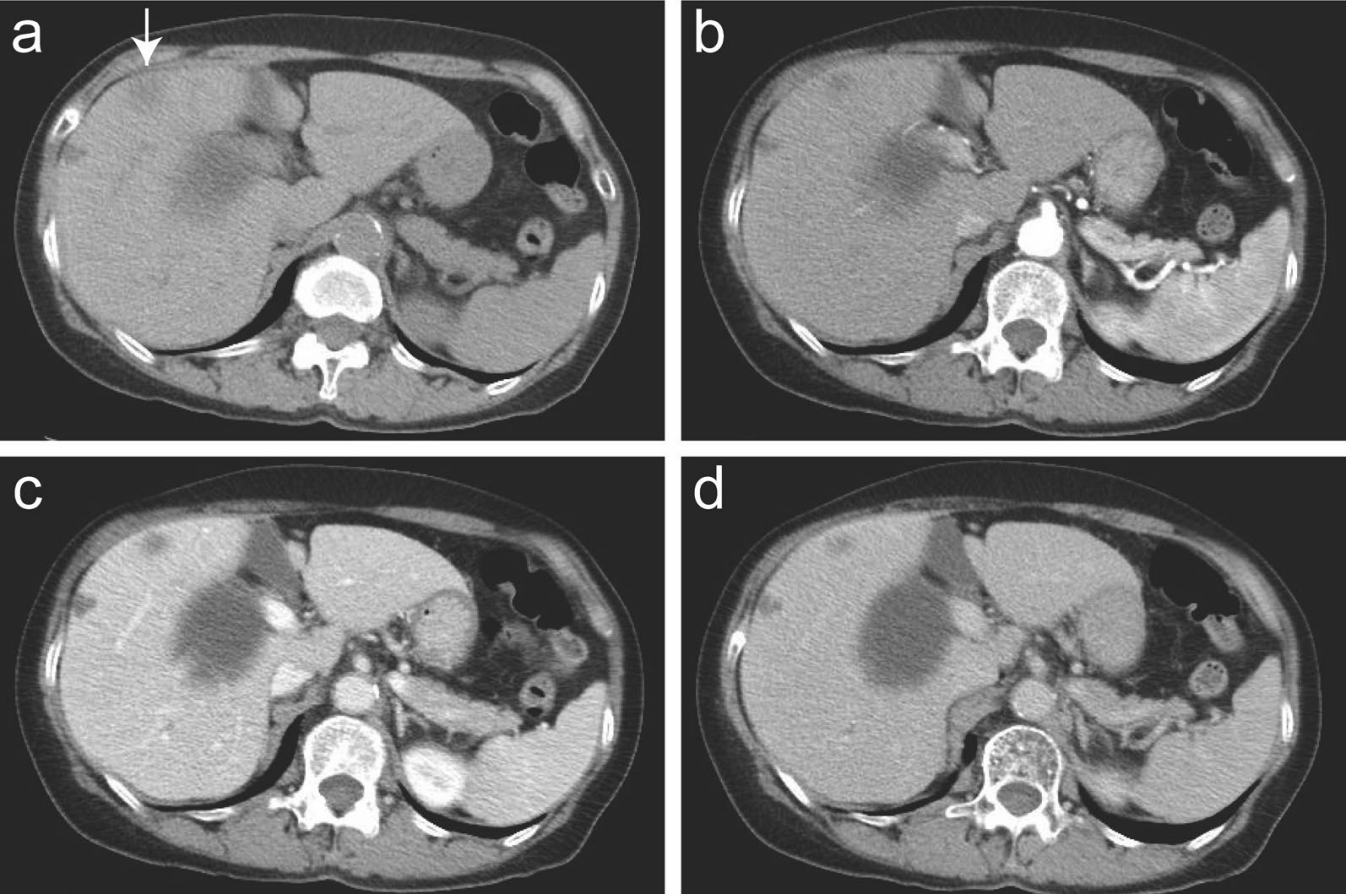
Contrast-enhanced dynamic computed tomography (CT). A plane CT scan showing an ambiguous and low-density mass (2.2 cm in diameter) in liver segment 8 (**a**). Arterial, portal, and venous phase CT scans showing fair marginal enhancement in all phases (**b**–**d**) and fair delayed enhancement in the inner area (**d**)

**Fig. 2:**
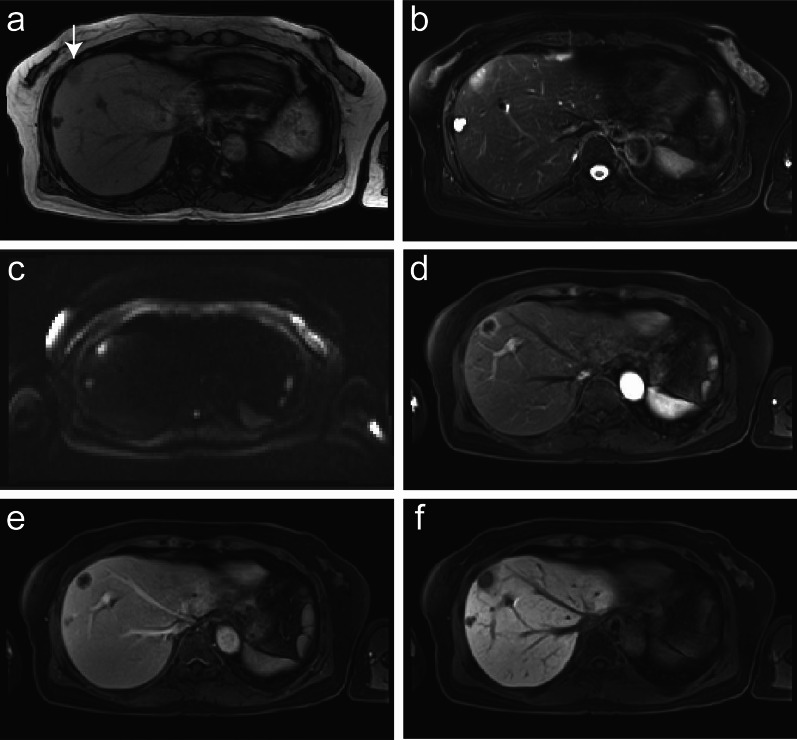
Magnetic resonance imaging (MRI) and gadolinium-ethoxybenzyl-diethylene-triaminepentaacetic acid (Gd-EOB-DTPA) MRI. MRI shows low intensity on T1-weighted (**a**) and high intensity on T2-weighted (**b**) and diffusion-weighted (DWI) images (**c**). Enhanced MRI shows marginal enhancement in the arterial phase (**d**) and venous phase (**e**) and hypointensity in the hepatocellular phase (**f**)

**Fig. 3:**
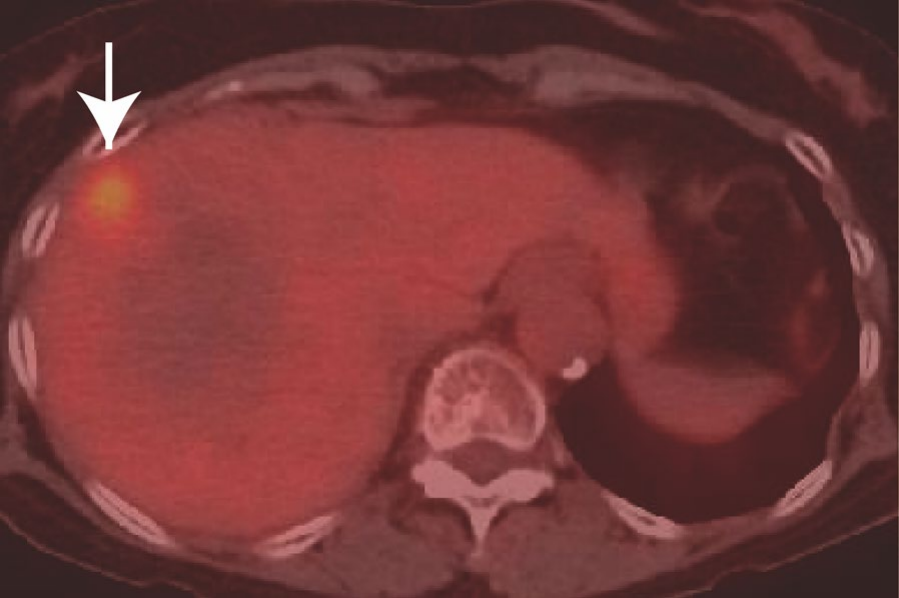
FDG-positron emission tomography (PET). PET shows high integration (SUVmax:5.9) in the tumor

The patient underwent 4 years and 6 months of follow-up surveillance according to the Japanese Society for Cancer of the Colon and Rectum (JSCCR) Guideline for the treatment of colorectal cancer. Serum tumor markers, which are recommended to be examined every 6 months after 3 years, remained within the normal ranges until 4 years and 6 months after ileocecal resection. She had been diagnosed with liver cysts but not liver tumors on an enhanced computed tomography (CT) scan until 5 years after ileocecal resection. However, due to the patient’s fatigue, serum examinations were canceled at 5 years. After persuading her to undergo the final follow-up examination three months later, the serum carbohydrate antigen 19-9 (CA19-9) level increased to 929 U/ml, not observed 9 months earlier (4.5 years after ileocecal resection), with no detectable mass lesions on abdominal ultrasound sonography (AUS). Other parameter levels were within normal ranges in the serum analysis. We suggested that the patient undergo an enhanced CT scan. However, she hesitated to accept our recommendation, expressing disappointment with the results, and declined further examinations and treatment. Two weeks later, she presented herself, and subsequent dynamic enhanced CT detected an ambiguous low-density tumor measuring 2.2 × 2 cm in liver segment 8 (Fig. 1a), and serum CA19-9 levels further increased to 2023 U/ml. The tumor exhibited delayed enhancement in the inner part and marginal enhancement in all phases (Fig. 1b–d). Peripheral intrahepatic bile duct dilatation and regional lymph node swelling were absent. Magnetic resonance imaging (MRI) revealed low intensity on T1-weighted images (Fig. 2a), high intensity on T2-weighted images (Fig. 2b), and high intensity on diffusion-weighted images (DWI) (Fig. 2c). Gadolinium-ethoxybenzyl-diethylenetriaminepentaacetic acid (Gd-EOB-DTPA) MRI showed marginal enhancement in the arterial-to-venous phase (Fig. 2d and e) and hypointensity in the hepatocellular phase (Fig. 2f). Fluorodeoxyglucose-positron emission tomography (FDG-PET) revealed high integration into the tumor (SUVmax, 5.9; Fig. 3) without any suspected metastasis or lymph node metastasis 10 days after tumor detection on CT. Tests for hepatitis B surface antigen and hepatitis C virus antibodies were negative. Further gastrointestinal workup, including upper and lower endoscopies, yielded negative results.

**Fig. 4:**
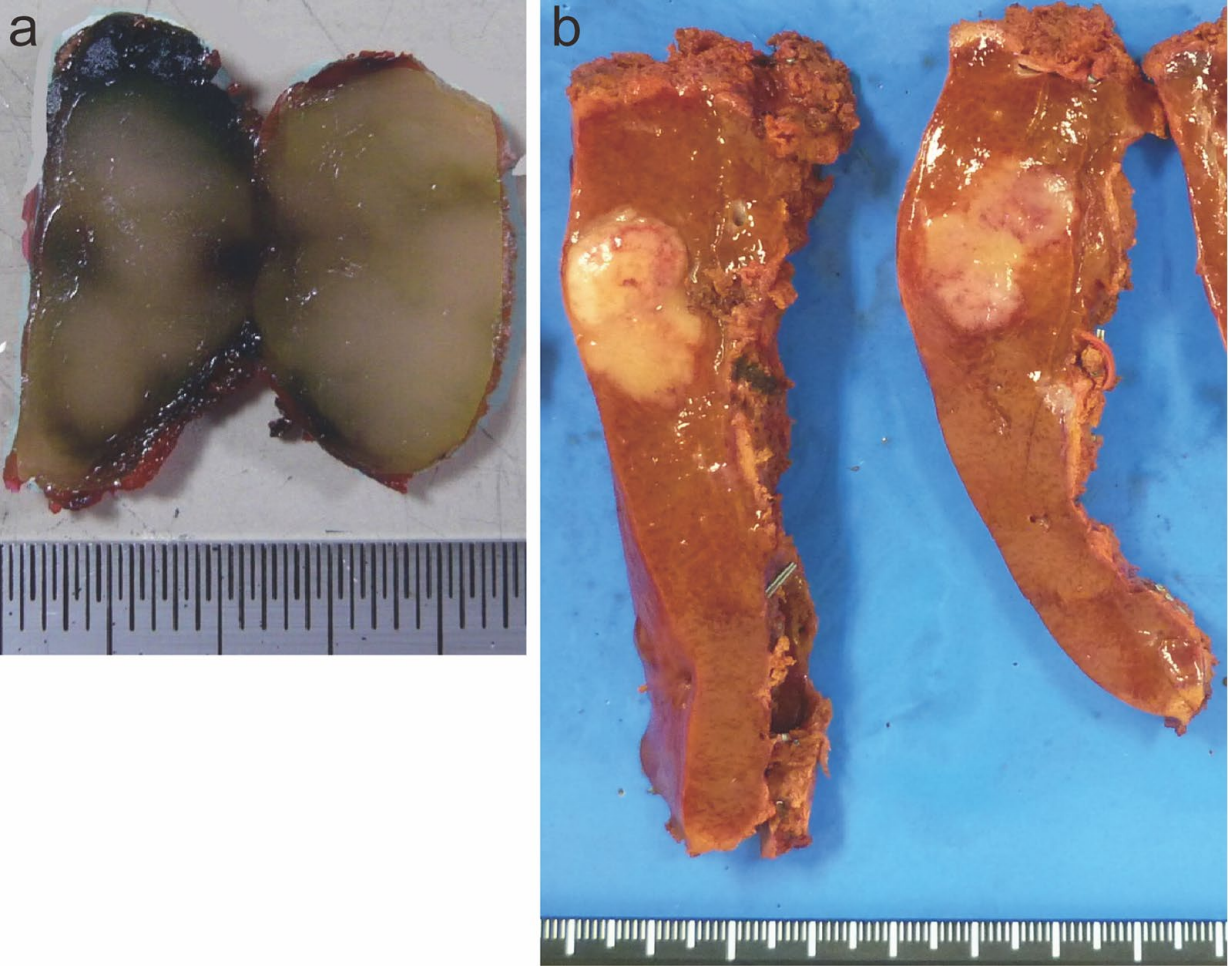
Gross findings of the resected specimen. Formalin-fixed lymph node after frozen rapid histopathologic diagnosis during surgery swells 3.0 × 1.5 × 1.2 cm (**a**). Liver tumor is fibrotic hard, yellowish-white, and solid measuring 3.0 cm × 2.5 cm × 2.0 cm with an irregular border, bleeding, and necrosis in inner area (**b**)

The patient was diagnosed with ICC or atypical metastatic tumor from past cecal cancer without lymph node or distant metastases and resection was planned 3 weeks after PET–CT was performed. Liver function in this patient was well-maintained at Child–Pugh A, and the indocyanine green retention rate at 15 min was 2.0%. The remnant liver volume after right anterior sectionectomy was 675 ml (63.6%). The patient was scheduled to undergo hepatectomy after informed consent was obtained. Despite the initial plan for right anterior sectionectomy, laparotomy revealed swelling of the hilar lymph node, measuring 3 × 1.5 cm (Fig. 4a). Since pathological examination during surgery revealed lymph node metastasis of adenocarcinoma, she underwent right anterior sectionectomy with hilar lymph node dissection. The operative time was 486 min, and the blood loss was 400 ml.

On gross findings, the liver specimen showed a fibrotic hard, yellowish-white, and solid mass measuring 3.0 cm × 2.5 cm × 2.0 cm with an irregular border and bleeding and necrotic area in inner zone (Fig. 4b). The tumor lacked a capsule but seemed well-defined from the surrounding normal liver.

**Fig. 5:**
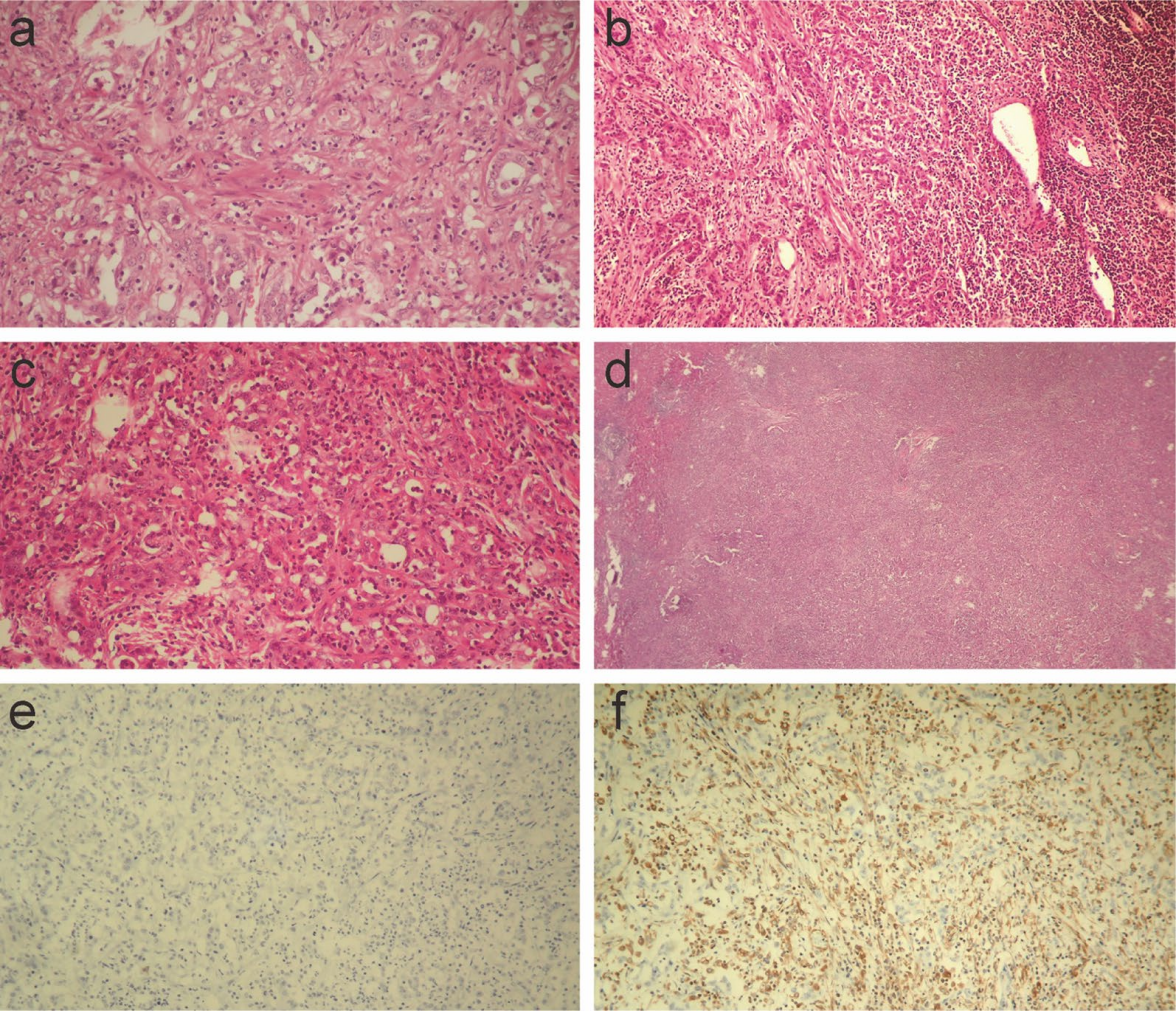
Histopathological examinations of liver tumor and lymph node. Poorly differentiated adenocarcinomas in the liver tumor (**a** H&E, magnification:100×) and lymph node (**b** H&E, magnification:40×) are shown. Liver tumor involves desmoplastic change. Pleomorphic or fusiform giant cells in the liver tumor (**c** H&E, magnification:100×) and obscure transition between adenocarcinoma and sarcomatous components (**d** H&E, magnification:20×) are shown. Immunohistochemical (IHC) examination shows desmin positivity (**e** magnification:100×) and vimentin negativity (**f** magnification:100×) in the liver tumor

Histopathological examination of both the liver tumor and lymph nodes revealed poorly differentiated adenocarcinomas (Fig. 5a and b). Although the lymph node specimen showed only adenocarcinoma (Fig. 5b), the liver tumor showed sarcomatous changes, including severe desmoplastic changes (Fig. 5a), and pleomorphic or fusiform giant cells (Fig. 5c). A large part of the tumor was covered by adenocarcinoma, which obscurely transited to a sarcomatous component covering approximately one-fifth of the tumor (Fig. 5d). The tumor had no histological elements suggesting HCC or bilirubin production. The surrounding liver parenchyma showed a normal liver.

**Fig. 6:**
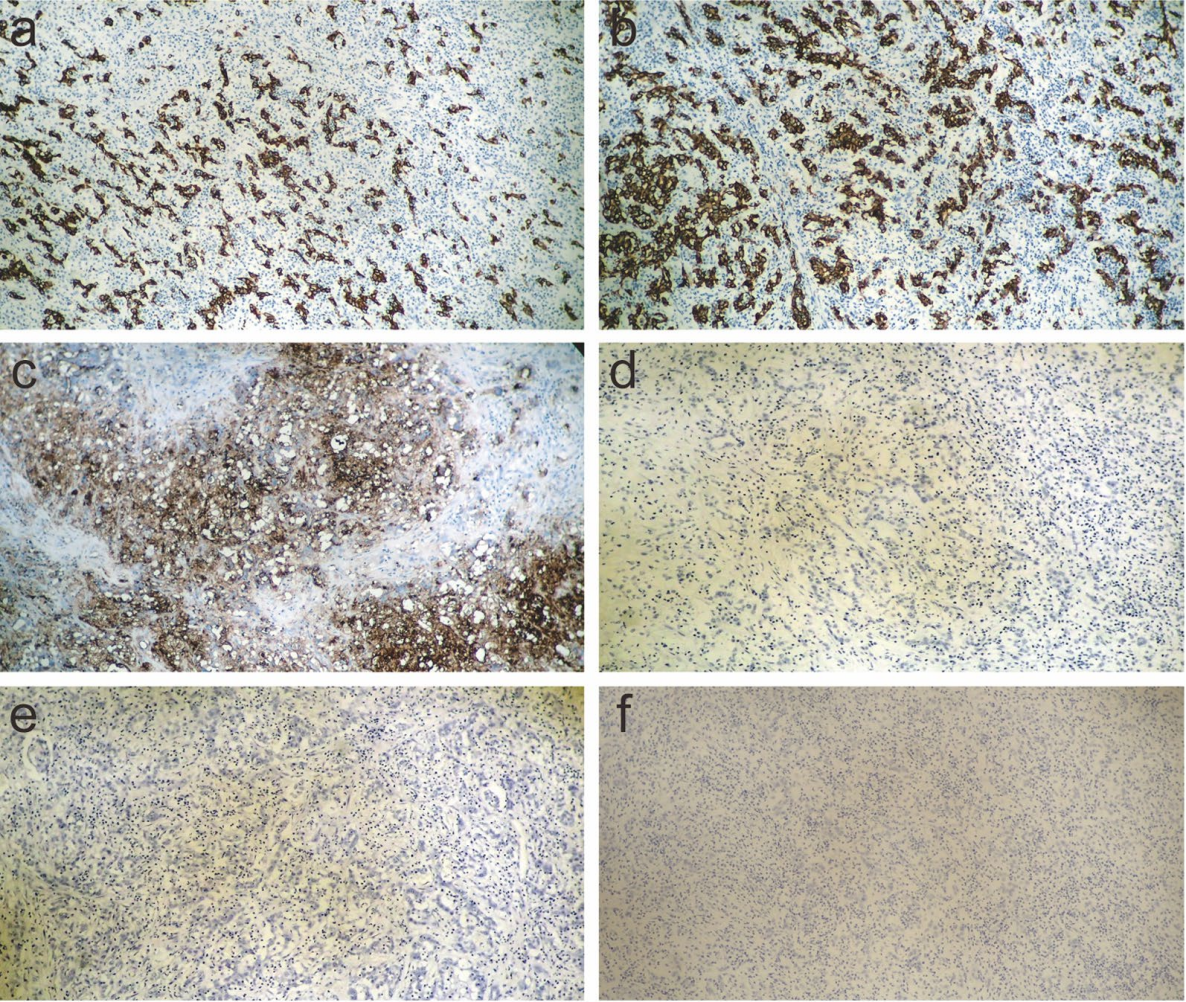
Immunohistochemical examination of liver tumor. Positive findings of cytokeratin-7 (CK7, **a**), CK19 (**b**), CA19-9 (**c**), and negative findings of CK20 (**d**), alfa-fetoprotein (AFP, **e**), carcinoembryonic antigen (CEA, **f**) are shown. (magnification:100×)

Immunohistochemical (IHC) analysis revealed that the cancer cells were negative for desmin (Fig. 5e) and positive for vimentin (Fig. 5f). The cancer cells were also positive for cytokeratin-7 (CK7; Fig. 6a), CK19 (Fig. 6b), and CA19-9 (Fig. 6c) but negative for CK20 (Fig. 6d), alpha-fetoprotein (AFP; Fig. 6e), and carcinoembryonic antigen (CEA, Fig. 6f). A negative finding for AFP and bilirubin production denied the possibility of CK19 positive HCC, and positive findings for CK7 with negative findings for CK20 and CEA denied the possibility of colorectal cancer metastasis. The final pathological diagnosis was sarcomatous ICC, and the tumor stage was determined to be T2N1M0 stage IVA, based on the 8th edition of the American Joint Committee on Cancer TNM staging system. The genomes of the tumors were microsatellite stable (MSS) and immunohistochemistry for mismatch repair gene deficiency (dMMR), including MLH1, MSH2, MSH6, and PMS2, was proficient in concluding that the tumor was MSS/MMR-proficient (pMMR).

The postoperative course was uneventful and the patient was discharged on postoperative day 15. Serum CA19-9 went down to a normal range after resection. She received a combination of gemcitabine and S-1 for six months as adjuvant therapy. Follow-up diagnostic imaging and blood examination showed lymph node swelling in the mediastinum and elevation of serum CA19-9 (72 U/ml) and CEA (6.7 ng/ml) at 13 months after surgery. The patient was diagnosed with lymph node recurrence and received combination therapy of gemcitabine, cisplatin, and S-1; however, she died 20 months after surgery.

## Discussion

Sarcomatous features of ICC are relatively rare and often lead to poor prognosis, with a median overall survival of < 6 months [9, 11]. SICC has been reported to represent approximately 4.5% of surgical and autopsied ICC cases [12], and others have reported that the occurrence rate of SICC in ICC was 1.6% or lower in 466 ICC cases at a single institution [13]. This number is significant, warranting special attention to this cancer with a poor prognosis.

SICC has been identified to have no relationship with viral infection or cirrhosis, and its pathogenesis remains uncertain due to the biphasic differentiation originating from the same pluripotent cancer stem cells or the redifferentiation of an immature multipotent carcinoma cell clone [8]. The morphological features of this histological type are characterized by the coexistence of adenocarcinoma and sarcomatous components, and their relative proportions varying from case to case [12]. The sarcomatous component consists of spindle cells arranged in sheets or bundles with oval or elongated hyperchromatic nuclei, occasional mitotic figures, and pleomorphic cells with adenoid structures [14]. Watanabe et al. reported the diagnostic criteria for histopathological and immunohistochemical examinations, including the coexistence of both components and the expression of the molecular features of both the mesenchyme and epithelium in the sarcomatous component [15]. Immunohistochemically, SICC is positive for cytokeratin, vimentin, and epithelial membrane antigen but negative for smooth muscle actin, S-100, and desmin [12, 13, 16, 17]. These criteria could establish a distinction from carcinosarcoma, which has only mesenchymal features, or cholangiocarcinoma with sarcomatoid transformation, which has only epithelial features in its sarcomatous component [15, 18]. Our case contained histopathological features of the coexistence of poorly differentiated adenocarcinoma and sarcomatous changes, including severe desmoplastic changes and pleomorphic-to-fusiform giant cells. These cancer cells were negative for desmin, but positive for vimentin, CK7, and CK19 on immunohistochemical examination. These results are consistent with the fact that sarcomatous transformation of epithelial cells retains some features of their original phenotype [19].

Our patient had poorly differentiated adenocarcinoma with bleeding and necrosis in the inner zones. These changes are generally observed in SICC because an insufficient neoangiogenic network usually induces poorly differentiated and rapidly growing sarcomatous cells that lack an adequate metabolic supply, resulting in wide necrotic areas [20]. Necrotic changes contribute to the radiological finding of a low-density lesion with a necrotic area in the inner part and peripheral contrast enhancement. This radiological behavior makes it difficult to distinguish SICC from atypical liver abscesses [14, 16, 21], metastatic tumors, or ICC [8, 22–26]. The ambiguous and non-descriptive imaging findings of SICC render preoperative diagnosis unreliable, often resulting in a missed clinical diagnosis [8, 27], a significant clinical issue associated with SICC. Secondly, SICC tends to grow rapidly at an astonishing rate, contributing to a poor prognosis [9]. While the survival rate of patients with resectable SICC treated with surgery alone is significantly higher than that of patients without surgical resection [28], it is as low as that of untreated ICC [15, 29]. However, consensus has not been reached on preoperative neoadjuvant chemotherapy (NAC) for resectable SICC or ICC. Additionally, preoperative biopsy, considered when a tumor cannot be diagnosed, poses the potential risk of peritoneal dissemination and takes time from sample collection to result explanation. Currently, it is deemed appropriate to perform upfront resection without preoperative biopsy in resectable SICC or ICC cases and to utilize surgical specimens for treatment in cases of recurrence. However, optimal diagnosis and treatment strategies for SICC are in high demand. Regarding preoperative chemotherapy, gemcitabine-based chemotherapy for locally advanced ICC demonstrated similar short- and long-term results to those of patients with initially resectable ICC [30]. The population of MSI-H was only 2.0% in cholangiocarcinoma [31], and the KEYNOTE-158 study demonstrated that cholangiocarcinoma patients with MSI-H/dMMR who experienced failure with prior therapy remarkably benefit from the immune-checkpoint inhibitor pembrolizumab [32]. The later showed that objective response rate (ORR) was 40.9% (95% CI 20.7 to 63.6) among 22 patients. Surprisingly, 7 of 22 patients showed partial response (PR) and 2 of 22 showed complete resection (CR). These results suggest the feasibility of conversion therapy or downstaging in patients with initially unresectable ICC and shed light on the efficacy of NAC for resectable ICC. Others have suggested the efficacy of various NAC regimens in ICC cases with poor prognosis, such as those with lymph node-positive or multiple tumors [33]. Possibly, this approach may be applicable to SICC, which accounts for a considerable proportion of ICC. A definitive preoperative diagnosis following biopsy is required to perform NAC. Since preoperative biopsy is time-consuming and NAC for resectable tumors potentially induces tumor progression, this strategy should be considered with discretion. Clinical trials are required to evaluate the safety of resectable ICC or SICC and the curative resection rates in unresectable cases.

Hilar lymph node dissection (LND) was performed for lymph node metastasis; however, the efficacy of LND is controversial. One of the third cases of resected ICC showed LN metastasis, which correlated with poorer prognosis [34–36]. Some reports have suggested that prophylactic LND does not contribute to the prognosis of ICC patients without LN metastasis but might be useful for diagnostic staging and exclusion of positive regional LN [37, 38]. Others have reported that LND does not benefit the survival of patients regardless of whether LN is positive or negative [39, 40]; however, its usefulness for nodal staging in these patients has been suggested [39]. An expert consensus statement suggested that nodal staging by LND provides additional prognostic information [41], and the latest 8th edition of the American Joint Committee on Cancer (AJCC) Staging Manual recommends more than six LN samplings to diagnose LN metastasis and downstage regional LN metastasis from stage IV to IIIB, suggesting curability in some LN-positive patients [42]. Recently, some groups have suggested that LND improves oncologic outcomes in specific subset [43–45]. This trend in the increased use of LND suggests the growing adoption of AJCC recommendations for the treatment of ICC. Further studies are required to investigate the role of routine LND in ICC.

Several studies have shown that gemcitabine, cisplatin, and a 5-fluorouracil based regimen as adjuvant chemotherapy prolongs disease-free survival [15, 19]. The ABC-02 trial in the UK and the BT-22 trial in Japan indicated that combination chemotherapy with gemcitabine and cisplatin is a potential treatment option for locally advanced or metastatic biliary tract cancer (BTC) and sarcoma [46, 47]. Based on the results of the FUGA-BT trial, a combination of gemcitabine and S-1 instead of cisplatin was used in these patients [48]. Furthermore, adjuvant S-1 was considered as the standard therapy for resected BTC in Asian patients in the ASCOT trial [49]. In view of the poorer prognosis in SICC than in BTC, our patients received a combination therapy of gemcitabine and S-1 for 6 months. After lymph node recurrence was proven at 13 months, combination therapy with gemcitabine, cisplatin, and S-1 was administered. Because the tumor was MSS/pMMR, the patient was ineligible for pembrolizumab. As our hospital is not certified to perform a gene panel examination, and she refused referral to a certified institution, a gene panel examination was not performed. Pemigatinib is an oral inhibitor of fibroblast growth factor (FGR) and demonstrated the therapeutic potential in patient with FGFR2 fusions or rearrangements [50]. Entrectinib is an inhibitor of tropomyosin receptor kinase (TRK) and showed clinically meaningful response in NTRK fusion-positive solid tumors including cholangiocarcinoma [51]. If these relevant gene mutations were present, the patient would have received pembrolizumab or relevant molecular-targeting therapies with insurance coverage. Other promising molecular targets and drugs are being developed and the results of clinical trials are awaited.

## Conclusions

In conclusion, we present an unusual case of sarcomatous intrahepatic cholangiocarcinoma that posed challenges in preoperative diagnosis and provide valuable insights into the tumor growth rate. Because SICC is often missed in clinical diagnosis, and rapid growth induces poor prognosis, a comprehensive treatment strategy is required. Although early diagnosis and resection are important to avoid reducing the chances of curative resection, survival with upfront surgery is not satisfactory. Alternatively, NAC following preoperative biopsy may potentially benefit ICC patients with a poor prognosis, including SICC. However, considering the rapid progression, as in this case, a strategy for introducing a preoperative biopsy should be cautious.

## Data Availability

Data sharing was not applied in this article, as no datasets were generated or analyzed during the current study.

## Abbreviations

ICC: Intrahepatic cholangiocarcinoma
HCC: Hepatocellular carcinoma
CA19-9: Carbohydrate antigen 19-9
CT: Computed tomography
MRI: Magnetic resonance imaging
FDG-PET: Fluorodeoxyglucose-positron emission tomography
UICC: Union for International Cancer Control
AUS: Abdominal ultrasound sonography
DWI: Diffusion-weighted imaging
Gd-EOB-DTPA: Gadolinium-ethoxybenzyl-diethylne-triaminepentaacetic acid
MSS: Microsatellite stable
dMMR: Mismatch repair gene deficiency
pMMR: MMR-proficient (pMMR)
CK: Cytokeratin
AFP: Alpha-fetoprotein
CEA: Carcinoembryonic antigen
AJCC: American Joint Committee on Cancer
NAC: Neoadjuvant chemotherapy
BTC: Biliary tract cancer
FGR: Fibroblast growth factor
TRK: Tropomyosin receptor kinase
